# Human voices communicating trustworthy intent: A demographically diverse speech audio dataset

**DOI:** 10.1038/s41597-025-05267-3

**Published:** 2025-05-31

**Authors:** Constantina Maltezou-Papastylianou, Reinhold Scherer, Silke Paulmann

**Affiliations:** 1 Department of Psychology and Centre for Brain Science, University of Essex, Colchester, CO4 3SQ UK; 2 Brain-Computer Interfaces and Neural Engineering Laboratory, School of Computer Science and Electronic Engineering, University of Essex, Colchester, CO4 3SQ UK

**Keywords:** Psychology, Research data

## Abstract

The multi-disciplinary field of voice perception and trustworthiness lacks accessible and diverse speech audio datasets representing diverse speaker demographics, including age, ethnicity, and sex. Existing datasets primarily feature white, younger adult speakers, limiting generalisability. This paper introduces a novel open-access speech audio dataset with 1,152 utterances from 96 untrained speakers, across white, black and south Asian backgrounds, divided into younger (N = 60, ages 18–45) and older (N = 36, ages 60+) adults. Each speaker recorded both, their natural speech patterns (i.e. “neutral” or no intent), and their attempt to convey their trustworthy intent as they perceive it during speech production. Our dataset is described and evaluated through classification methods between neutral and trustworthy speech. Specifically, extracted acoustic and voice quality features were analysed using linear and non-linear classification models, achieving accuracies of around 70%. This dataset aims to close a crucial gap in the existing literature and provide additional research opportunities that can contribute to the generalisability and applicability of future research results in this field.

## Background & Summary

The way we speak has been the subject of interdisciplinary research for decades, given its pivotal role in everyday interactions and its contribution to our survival and societal integration. Voice plays a vital role in human existence by facilitating expression, fostering connections, and conveying emotions and intentions^1^. Moreover, it enables individuals to perceive and interpret the expressions of others, including personality traits like trustworthiness^2^.

In the area of voice acoustics, the use of recorded speech audio samples has become fundamental^3–5^. Different datasets enable scientists to examine the intricacies of voice perception and cognition, emotion recognition, and listener predispositions and personality perceptions of a speaker, among other factors^3–5^. By leveraging such voice samples, we can enhance our understanding of human communication as well as contribute to the advancement of speech technologies that have seamlessly become part of everyday life^6,7^. Table 1 provides a summary of the speech acoustics examined in this paper.

**Table 1:** Summary characteristics of speech acoustics examined.

Acoustic signal	Measured in	Key characteristics
Fundamental frequency (F0); perceived as pitch.	Hertz (Hz)	F0 is the lowest rate of vocal fold vibrations, and vocal intonation is usually captured by F0 variability within an utterance.
Amplitude; perceived as loudness.	Decibels (dB)	Indicative of air pressure variations.
Harmonics-to-noise ratio (HNR)	dB	Lower HNR signifies more noise in a voice signal^34,35^. Noise in terms of voice, encompasses any component of the signal that interferes with the clarity, purity and overall quality of the intended speech signal. Typically, this noise is not harmonically related to the fundamental frequency of the voice, such as alterations in vocal fold tissue, muscle tension, respiratory patterns, or even ambient sounds and electronic interference^35^.
Jitter	%	Reveals micro-fluctuations in pitch caused by irregular vocal fold vibrations^3,36,37^. A lower percentage value indicates that there is a small variation in pitch frequency during speech production.
Shimmer	dB	Measures micro-fluctuations in amplitude, reflecting variations in voice intensity^3,36,37^.
Cepstral peak prominence (CPP)	dB	A lower CPP is indicative of a breathy voice^38–40^.
Long-term average spectrum (LTAS)	dB	A lower LTAS often indicates longer vocal tract sizes^18,39,41,42^, linked to deeper, more resonant voices associated with dominance, particularly observed in males^43,44^.

Re-using validated and standardised voice samples can assist researchers in conducting meaningful comparisons across studies. When we refer to “standardised” we mean voice samples that adhere to consistent and predefined stimuli characteristics such as audio file formats, sampling rates and spoken content across speakers. This practice leads to more reliable insights and advancements in the field. However, current research on voice trustworthiness tends to rely on younger, white western populations^3–5,8,9^. Focusing primarily on white, western populations can affect the generalisability of such outcomes and miss out on additional insights that could be gained from ethnic cross-examination, sometimes referred to as white western individualist bias (WWIB)^10^. Moreover, current research has predominantly focused on how *listeners* perceive speakers as trustworthy, rather than how *speakers* attempt to communicate trustworthy intent during speech production^3–5,8^. To enhance research opportunities and provide a broader, more diverse range of stimuli, we have created a unique speech audio dataset. This dataset embodies a diverse range of sentences, incorporating recordings from untrained speakers (i.e. not relying on actors) across various age-groups (i.e. age range of 18–90), sex, and ethnic (i.e. white, black, south Asian) backgrounds. Moreover, it encompasses both natural speech patterns and deliberate attempts to communicate trustworthiness within each spoken utterance, as perceived by the speakers.

This paper describes our speech audio dataset, focusing on speaker demographics in relation to their intent to sound trustworthy versus their natural speaking voice, termed “neutral” intent. We validate the dataset as to how well the acoustic features can classify trustworthy intent to understand how these speakers attempt to convey trust based on their subjective perceptions, addressing a gap in the existing literature.

## Methods

### Ethics declaration

All procedures performed in this study were approved by the Ethics Subcommittee 2 of the University of Essex (ETH2324-2113) and were carried out in accordance with the Declaration of Helsinki. All participants provided informed consent prior to participation, where they were also briefed that their anonymised voice recordings, ratings and overall data could be (1) shared in publicly accessible archives and (2) used in future research studies.

### Participants

Ninety-six untrained (i.e. not actors), English-speaking adults were recruited to record the audio stimuli. All younger adult speakers (all below 45 years of age), and older (all 60 years or older) white speakers were recruited online through Prolific^11^, an online participant recruitment panel. Most older black and older south Asian speakers were recruited through posters and word of mouth given the lack of responses on Prolific. See Table 2 for more details on speaker demographics. We opted for a maximum age of 45 for younger adults, in an attempt to have a wide-enough age gap between younger and older speakers. All speakers reported normal hearing and were given a monetary reward. Throughout this paper, the terms participants/speakers may be used interchangeably.

**Table 2:** Descriptive statistics of speaker demographics.

Ethnicity	Age-group	Sex	Mean age (years)	Age range (years)	SD
White	Younger	Female (n = 10)	31.70	21–44	7.99
		Male (n = 10)	29.70	21–43	6.31
	Older	Female (n = 10)	70	60–87	9.51
		Male (n = 10)	67	60–76	5.85
Black	Younger	Female (n = 11)	27.64	22–42	5.70
		Male (n = 9)	29.22	20–37	6.36
	Older	Female (n = 5)	61	60–62	0.71
		Male (n = 3)	61.33	60–63	1.53
South Asian	Younger	Female (n = 10)	29	22–39	5.70
		Male (n = 10)	28.20	18–40	6.12
	Older	Female (n = 4)	66.75	60–77	7.63
		Male (n = 4)	70.50	61–90	13.33

### Materials

The materials were designed to not bias towards a specific emotional reading (e.g. You may call me anytime), as to not influence or bias the listener with loaded language or emotional tone. They were also controlled for sentence length, resulting in twenty 7-syllable sentences. A full list of the sentences created can be found in Table 3.

**Table 3:** All 20 sentences spoken in the speech audio dataset.

Number/Code	Sentence
1	I can drive you if you want.
2	You may use my car later.
3	Hello, I arrived early.
4	I will give you a lift home.
5	You should visit more often.
6	I can remind you later.
7	You may bring a friend with you.
8	I will save a seat for you.
9	I will direct you on this.
10	Hi, the shops are still open.
11	Hi, I’m waiting for someone.
12	You should wear something warmer.
13	You may call me anytime.
14	I will call you a taxi.
15	You should call me tomorrow.
16	You should get to know the team.
17	I can send you a message.
18	I can give you some guidance.
19	Hello, welcome to the team.
20	You may borrow these two books.

### Recording procedure

The recording process occurred online via a project-specific website, with participants primarily engaging remotely. However, one older adult was recorded in person due to a lack of computer access. Participants recorded their allocated materials using their personal computers and microphones. To mitigate the lack of control over the recording environment, speakers were instructed to record their voice in front of a computer that has a working microphone, in a quiet room with no background noise or other people talking or interfering, and to minimise interruptions (e.g., turn off phones). This approach follows past research from online versus lab-based studies^4,12,13^.

Participants were asked to speak all sentences assigned to them twice: first, in their natural tone of voice (i.e. neutral intent), and then, with the intention of eliciting trust from the listener (i.e. trustworthy intent). To mitigate experimenter bias, no examples were provided on how they should sound. A researcher was present remotely during each recording to answer any queries, observe whether the instructions had been followed appropriately and assess the quality of the recordings to mark completion. Each participant submitted an audio file containing at least twelve utterances.

### Audio pre-processing

#### Sampling rate and file format standardisation

Audacity audio editing and recording software (version 2.3.3) was used to standardise all recordings at a sampling rate of 48.0 kHz, 16-bits depth and 768 kb/s bit rate using a mono channel. The audio files were stored in an uncompressed WAV format.

#### Segmentation and intensity normalisation

Praat software (version 6.2.16)^14^ was used to segment all WAV files. Subsequently, each shorter sound file (i.e. sentence) was evaluated to eliminate any potential duplicates and normalised to 67 dB. Therefore, a total of 1,152 audio files (576 neutral and 576 with trustworthy intent) are accounted for in the final speech audio dataset.

#### Acoustic and spectral feature extraction

All acoustic and spectral features were extracted using VoiceLab software to analyse multiple audio files at once^15,16^. The features used in the analyses to describe the materials are mean F0 for perceived pitch, standard deviation of F0 for perceived pitch variability, sentence duration, HNR, jitter, shimmer, CPP, LTAS, standard deviation of the LTAS and LTAS slope. For our analyses, VoiceLab’s auto-correlation values were used for F0, the relative average perturbation (RAP) value for jitter, and the amplitude perturbation quotient 3 (APQ3) value for shimmer, as seen in past research^3,4^. Summary descriptives of each feature per intent can be found in Table 4 for white speakers, Table 5 for black speakers and Table 6 for south Asian speakers, while a definition of each acoustic can be found in Table 1.

**Table 4:** White speakers: Descriptive statistics of acoustic features per speaker intent, age-group and sex.

Acoustic features	Mean acoustic values [Standard deviation] for white speakers							
	Neutral intent				Trustworthy intent			
	Younger female	Younger male	Older female	Older male	Younger female	Younger male	Older female	Older male
*Duration (s)*	1.57 [0.31]	1.55 [0.36]	1.88 [0.36]	1.67 [0.29]	1.63 [0.39]	1.40 [0.30]	1.95 [0.48]	1.68 [0.39]
*F0, mean pitch (Hz)*	194.11 [18.55]	105.11 [14.24]	181.89 [24.94]	110.68 [20.72]	224.02 [24.38]	137.35 [31.63]	207.90 [27.29]	134.23 [30.31]
*F0, SD pitch (Hz)*	29.49 [15.84]	17.57 [12.00]	34.05 [16.17]	18.33 [14.92]	48.13 [19.10]	38.56 [24.65]	51.24 [18.15]	34.93 [18.55]
*HNR (dB)*	10.21 [2.62]	5.10 [2.27]	10.81 [2.63]	6.15 [1.43]	10.76 [2.74]	4.18 [2.09]	11.12 [2.28]	4.60 [1.87]
*Jitter (RAP)*	0.01 [0.00]	0.01 [0.00]	0.01 [0.00]	0.01 [0.00]	0.01 [0.00]	0.01 [0.00]	0.01 [0.00]	0.01 [0.00]
*Shimmer (APQ3)*	0.04 [0.02]	0.04 [0.01]	0.04 [0.01]	0.06 [0.02]	0.04 [0.02]	0.05 [0.01]	0.04 [0.01]	0.06 [0.02]
*CPP (dB)*	28.20 [2.40]	25.57 [2.19]	27.69 [2.19]	24.99 [2.05]	28.64 [2.41]	25.16 [2.63]	27.93 [2.44]	24.70 [2.21]
*LTAS, mean (dB)*	−1.51 [6.37]	−5.42 [7.80]	−2.92 [4.92]	−7.78 [6.96]	−2.46 [6.38]	−5.75 [8.36]	−3.51 [4.99]	−7.95 [7.43]
*LTAS, SD (dB)*	17.27 [2.20]	18.79 [3.28]	16.67 [1.31]	18.05 [2.97]	17.53 [2.06]	18.82 [3.33]	16.95 [1.36]	18.55 [3.11]
*LTAS slope (dB/octave)*	−13.16 [4.03]	−14.41 [4.54]	−15.98 [3.83]	−17.51 [4.01]	−12.58 [3.39]	−13.78 [4.53]	−16.58 [3.81]	−17.13 [4.28]

**Table 5:** Black speakers: Descriptive statistics of acoustic features per speaker intent, age-group and sex.

Acoustic features	Mean acoustic values [Standard deviation] for black speakers							
	Neutral intent				Trustworthy intent			
	Younger female	Younger male	Older female	Older male	Younger female	Younger male	Older female	Older male
*Duration (s)*	1.58 [0.26]	1.66 [0.46]	2.21 [0.55]	1.61 [0.25]	1.56 [0.29]	1.46 [0.36]	2.01 [0.51]	1.63 [0.31]
*F0, mean pitch (Hz)*	174.35 [23.94]	110.49 [22.11]	176.64 [32.06]	101.44 [13.79]	211.98 [23.32]	129.81 [21.96]	220.49 [46.00]	140.45 [48.16]
*F0, SD pitch (Hz)*	29.43 [16.15]	15.58 [9.80]	28.90 [14.15]	14.89 [12.12]	41.83 [16.02]	25.18 [14.13]	52.82 [21.14]	34.30 [36.34]
*HNR (dB)*	10.47 [2.91]	6.36 [2.19]	10.85 [3.22]	5.76 [3.29]	10.54 [2.78]	5.90 [2.52]	10.29 [3.01]	6.83 [2.67]
*Jitter (RAP)*	0.01 [0.00]	0.01 [0.01]	0.01 [0.00]	0.01 [0.00]	0.01 [0.00]	0.01 [0.00]	0.01 [0.00]	0.01 [0.00]
*Shimmer (APQ3)*	0.04 [0.01]	0.04 [0.01]	0.03 [0.01]	0.04 [0.01]	0.03 [0.01]	0.04 [0.01]	0.03 [0.01]	0.04 [0.01]
*CPP (dB)*	26.93 [2.18]	25.77 [2.18]	27.10 [2.76]	23.53 [1.83]	27.61 [2.10]	25.80 [2.44]	26.97 [3.29]	24.30 [1.68]
*LTAS, mean (dB)*	−3.15 [5.47]	−5.54 [7.63]	−12.72 [8.25]	−19.27 [7.03]	−2.96 [5.41]	−6.72 [8.15]	−14.05 [7.48]	−20.46 [4.02]
*LTAS, SD (dB)*	17.10 [1.76]	17.68 [2.60]	22.39 [5.13]	25.17 [2.31]	17.55 [1.74]	17.80 [2.73]	23.59 [5.21]	26.07 [1.97]
*LTAS slope (dB/octave)*	−15.48 [4.09]	−15.47 [5.28]	−16.50 [3.39]	−15.21 [5.49]	−13.76 [4.01]	−15.21 [4.66]	−13.87 [4.47]	−15.85 [5.03]

**Table 6:** South Asian speakers: Descriptive statistics of acoustic features per speaker intent, age-group and sex.

Acoustic features	Mean acoustic values [Standard deviation] for south Asian speakers							
	Neutral intent				Trustworthy intent			
	Younger female	Younger male	Older female	Older male	Younger female	Younger male	Older female	Older male
*Duration (s)*	1.59 [0.28]	1.56 [0.27]	1.96 [0.41]	1.85 [0.46]	1.45 [0.25]	1.48 [0.27]	1.85 [0.48]	2.05 [0.72]
*F0, mean pitch (Hz)*	189.60 [25.73]	119.75 [14.40]	189.63 [12.66]	135.66 [43.21]	230.60 [35.62]	135.08 [22.65]	224.19 [40.35]	155.86 [36.61]
*F0, SD pitch (Hz)*	31.30 [15.05]	21.29 [12.15]	30.86 [10.66]	25.21 [29.67]	47.72 [18.98]	30.65 [20.10]	50.29 [14.05]	40.23 [23.73]
*HNR (dB)*	12.06 [3.22]	7.62 [3.29]	12.07 [1.98]	6.74 [3.37]	11.62 [2.98]	7.44 [2.98]	11.38 [3.05]	6.63 [3.92]
*Jitter (RAP)*	0.01 [0.00]	0.01 [0.00]	0.01 [0.00]	0.01 [0.00]	0.01 [0.01]	0.01 [0.01]	0.01 [0.01]	0.01 [0.00]
*Shimmer (APQ3)*	0.04 [0.01]	0.05 [0.02]	0.04 [0.01]	0.05 [0.02]	0.03 [0.01]	0.05 [0.02]	0.04 [0.01]	0.05 [0.02]
*CPP (dB)*	27.38 [2.59]	25.49 [2.51]	27.95 [1.95]	26.07 [1.53]	27.37 [3.22]	24.76 [2.55]	28.06 [2.53]	27.42 [2.54]
*LTAS, mean (dB)*	−6.67 [10.32]	−8.62 [8.34]	−11.75 [10.01]	−9.09 [4.06]	−7.66 [10.44]	−8.06 [8.43]	−12.89 [9.50]	−8.72 [4.95]
*LTAS, SD (dB)*	16.96 [2.38]	16.30 [3.85]	17.26 [5.75]	18.74 [3.74]	16.97 [2.48]	16.93 [3.54]	17.55 [6.28]	18.60 [3.34]
*LTAS slope (dB/octave)*	−18.22 [4.91]	−19.71 [6.59]	−19.69 [5.18]	−14.79 [4.65]	−17.69 [5.67]	−18.38 [6.87]	−18.37 [7.40]	−15.74 [3.19]

## Data Records

The speech audio dataset is publicly available on the Open Science Framework (OSF) repository^17^ (10.17605/OSF.IO/45D8J) under the CC-by Attribution 4.0 International license. All data are anonymous, and available in a folder named “Speaker Data”. Inside this folder two CSV files can be found containing speaker demographics and extracted acoustic features per speech audio file. There is also a “README.md” file, which offers additional guidance on how to find and make use of the current dataset. There are also two sub-folders:

### “Speech WAV Files”

This sub-folder contains all 1,152 speech audio recordings of our dataset in.wav format, normalised to 67 dB. The audio files are further split into sub-folders by speaker ethnicity and age group. The name of each audio file follows the sequence of “speaker ID”_“ethnicity” “age-group” “sex”_“intent” “sentence number”. For example, the filename “1901_bof_t05.wav” indicates that this file has been recorded by speaker ID 1901 of black (b), older (o) and female (f) demographic background who has used a trustworthy (t) intent when speaking sentence #5 (i.e. “You should visit more often”). The audio file 1901_bof_n05.wav is from the same speaker, speaking the same exact sentence but in this instance, they have used their natural speaking voice (i.e. neutral “n” intent). See Table 7 for more information.

**Table 7:** Dataset’s audio file name abbreviations.

Speaker	Abbreviation	Audio filename examples
White	w	1893_**w**of_t05.wav
Black	b	1901_**b**of_t05.wav
South Asian	a	2017_**a**of_t05.wav
Younger	y	1906_b**y**f_t05.wav
Older	o	1901_b**o**f_t05.wav
Male	m	2233_bo**m**_t05.wav
Female	f	1901_bo**f**_t05.wav
Neutral	n	1901_bof_**n**05.wav
Trustworthy	t	1901_bof_**t**05.wav

### “Python_SourceCode_SpeechDB”

This sub-folder contains a .txt file listing all relevant Python package dependencies with their respective versions, and a “Scripts” sub-folder containing the “main.py” file for running the analyses seen in the Technical Validation section of this paper.

## Technical Validation

Our recordings relied on speakers’ intention to convey trustworthiness. To evaluate whether the captured voice samples exhibit measurable differences between neutral speech and speech with a trustworthy intent, we analysed a set of commonly used acoustic and spectral features^18–20^ – see also Table 1. These features were then used as input to classifiers to determine whether successful classification was possible, thereby validating the presence of measurable acoustic differences between the two speech intent conditions. Specifically, the speech audio dataset has been validated using established classification methods, i.e. Random Forest (RF)^21–25^ and Logistic Regression (LR)^21,26,27^. We have investigated how trustworthy intentions during speech production relate to acoustic features across demographically diverse speakers. As the data were recorded in real-life settings outside a controlled lab environment, they may include technical variations such as differing microphone qualities and noise levels. While these variations were anticipated, they reflect the practical challenges of data collection in non-controlled environments.

To handle the complexities of our dataset (i.e. extracted acoustic features, diverse ethnic and age groups, speaker intent), a RF classification algorithm (126 trees; random state with a value of 1 for reproducibility purposes) was chosen for its ability to handle multi-dimensional data and robustness to noise. Moreover, RF enhances generalisability by aggregating predictions from multiple independent hierarchical models known as decision trees, and includes a built-in measure of feature importance (i.e. can assess the contribution rate of each acoustic feature towards the classification between trustworthy and neutral intents).

To further evaluate the robustness of the RF model’s classification accuracy, we compared its results with another model, namely logistic regression (random state with a value of 1). For each classification method, we have employed a leave-one-speaker-out cross-validation (LOSO CV) strategy^28,29^. The added benefit of LOSO CV stems from the fact that it has allowed us to validate our models more thoroughly by assessing the model’s sensitivity in discriminating trustworthy from neutral intent considering individual speaker idiosyncrasies.

### Trustworthy intent classification

All extracted acoustic features have been used in both LR and RF models. As seen in Table 8, the overall (i.e. all data included) performance in detecting trustworthy speech, revealed similar metric scores between the two models. When splitting the data by ethnicity, some variation has been noted for black and south Asian ethnicities for both models. This variation may possibly be due to the unbalanced number of participants recruited per age-group for those two ethnicities in the dataset, considering that the white ethnic group and independent assessment of each age-group have gained better performance. See Table 9 for the confusion matrices results.

**Table 8:** LOSO CV classification results: Comparison of RF and LR trustworthy intent.

Data	Random Forest				Logistic Regression			
	Accuracy	Precision	Recall	F1 Score	Accuracy	Precision	Recall	F1 Score
**Overall**	71%	73%	68%	70%	69%	71%	66%	68%
**Per Ethnicity**								
White	71%	70%	73%	71%	72%	73%	69%	71%
Black	68%	69%	66%	68%	68%	69%	66%	67%
South Asian	66%	68%	61%	64%	68%	69%	64%	66%
**Per Age-group**								
Younger adults	70%	71%	66%	68%	67%	69%	63%	66%
Older adults	69%	71%	66%	68%	72%	73%	69%	71%

**Table 9:** Confusion matrices results: Comparison of RF and LR trustworthy intent.

Data	Random Forest					Logistic Regression			
	True Positives	False Positives	True Negatives	False Negatives		True Positives	False Positives	True Negatives	False Negatives
**Overall**	394	148	428	182		378	157	419	198
**Per Ethnicity**									
White	174	73	167	66		165	60	180	75
Black	111	49	119	57		111	51	117	57
South Asian	102	48	120	66		107	47	121	61
**Per Age-group**									
Younger adults	237	96	264	123		227	104	256	133
Older adults	142	58	158	74		150	56	160	66

Moreover, we have evaluated these models through the Receiver Operating Characteristic (ROC) curves and compared the Area Under the Curve (AUC) values. The ROC curve illustrates classifier performance, while the AUC score from 0–1 (where 1 = perfect classifier) quantifies its ability to distinguish trustworthy from neutral intent (see Table 10). Both RF and LR models have reliably exhibited above average classification performance (RF AUC values between 71–77%; LR AUC values between 72–78%).

**Table 10:** AUC values: Comparison of RF and LR trustworthy intent.

Data	Random Forest AUC values	Logistic Regression AUC values
**Overall**	77%	76%
**Per Ethnicity**		
White	77%	78%
Black	71%	74%
South Asian	73%	72%
**Per Age-group**		
Younger adults	75%	75%
Older adults	75%	76%

### Acoustic feature importance

**Fig. 1:**
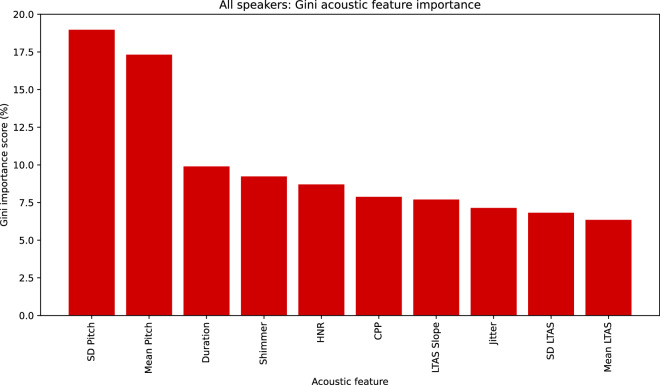
Common Gini feature importance across all speaker demographics: RF acoustic feature contribution in % towards the classification of trustworthy intent. Classification accuracy was 71%.

**Fig. 2:**
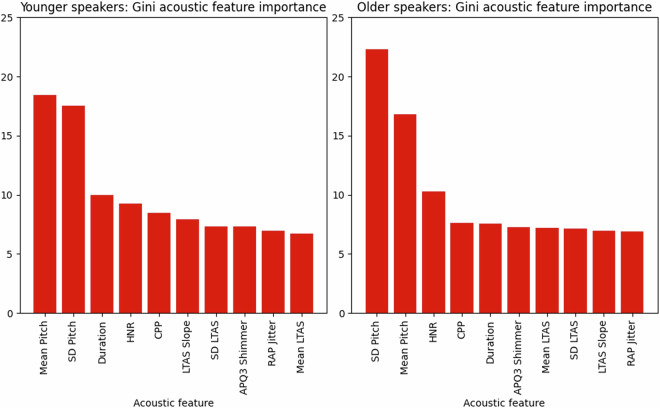
RF acoustic feature contribution in % towards the classification of trustworthy intent, by speaker age-group.

**Fig. 3:**
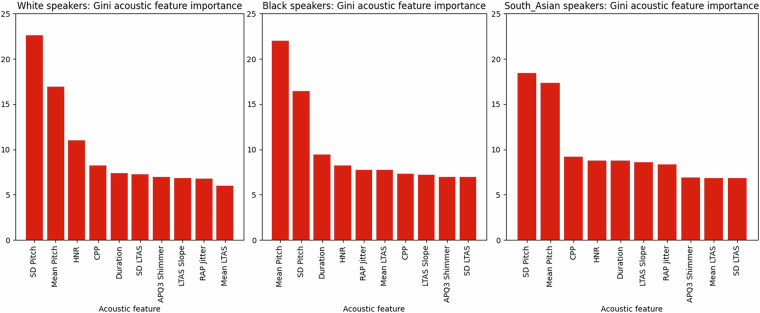
RF acoustic feature contribution in % towards the classification of trustworthy intent, by speaker ethnicity.

We have applied the Gini feature importance function as part of our RF analysis to delineate the contribution of each extracted acoustic feature towards the classification of trustworthy speaker intent – common across all speaker demographics (see Fig. 1 for the Gini output), as well as separately per ethnicity and age-group (see Figs. 2, 3 for the Gini output). The Gini feature importance figures can be seen side by side for comparison with the LR acoustic significance findings (see Tables 11–13). Pitch, HNR, shimmer and CPP seem to be the common contributors across all speaker demographics, albeit HNR appears more prominently for LR. Moreover, significant acoustics seem to vary between models and individual demographics, with yet again the most common leaning towards, pitch and HNR. LTAS seems to be consistently low in terms of feature importance in the RF model. Overall, both models seem to offer similar observations in terms of acoustic significance towards the classification of trustworthy speaker intent. They seem to align with and offer additional insights to past research examining these acoustic features^3,8,30–33^.

**Table 11:** Common acoustic significance across all speaker demographics: LR acoustic feature contribution towards the classification of trustworthy intent. Classification accuracy was 69%.

Acoustics	Coef. (β)	S.E.	z	p-value	95% C.I.		Odds Ratio (Exp(β))
					Lower	Upper	
*Duration*	−0.27	0.18	−1.50	0.13	−0.62	0.08	0.77
*F0, mean pitch*	0.02	0.00	7.44	**0**.**00**	0.02	0.03	1.02
*F0, SD pitch*	0.03	0.01	5.83	**0**.**00**	0.02	0.04	1.03
*HNR*	−0.27	0.04	−7.76	**0**.**00**	−0.34	−0.20	0.76
*Jitter, RAP*	−38.33	20.52	−1.87	0.06	−78.54	1.89	0.00
*Shimmer, APQ3*	−13.05	5.85	−2.23	**0**.**03**	−24.52	−1.59	0.00
*CPP*	−0.06	0.03	−1.73	0.08	−0.12	0.01	0.95
*LTAS, mean*	−0.03	0.01	−2.12	0.03	−0.05	0.00	0.97
*LTAS, SD*	−0.07	0.03	−2.14	**0**.**03**	−0.13	−0.01	0.93
*LTAS, slope*	0.00	0.02	−0.18	**0**.**86**	−0.04	0.03	1.00

**Table 12:** LR acoustic feature contribution towards the classification of trustworthy intent, by speaker age-group.

Acoustics	Coef. (β)	S.E.	z	p-value	95% C.I.		Odds Ratio (Exp(β))
					Lower	Upper	
**Younger adults**							
*Duration*	−0.65	0.30	−2.20	**0**.**03**	−1.23	−0.07	0.52
*F0, mean pitch*	0.02	0.00	6.53	**0**.**00**	0.02	0.03	1.02
*F0, SD pitch*	0.03	0.01	4.16	**0**.**00**	0.01	0.04	1.03
*HNR*	−0.30	0.05	−6.36	**0**.**00**	−0.39	−0.21	0.74
*Jitter, RAP*	−32.80	25.27	−1.30	0.19	−82.33	16.73	0.00
*Shimmer, APQ3*	−19.76	8.40	−2.35	**0**.**02**	−36.22	−3.31	0.00
*CPP*	−0.03	0.04	−0.82	0.41	−0.11	0.05	0.97
*LTAS, mean*	−0.04	0.02	−2.66	**0**.**01**	−0.08	−0.01	0.96
*LTAS, SD*	−0.15	0.05	−3.17	**0**.**00**	−0.25	−0.06	0.86
*LTAS, slope*	0.02	0.02	0.72	0.47	−0.03	0.06	1.02
**Older adults**							
*Duration*	−0.03	0.26	−0.13	0.90	−0.54	0.48	0.97
*F0, mean pitch*	0.02	0.01	3.35	**0**.**00**	0.01	0.03	1.02
*F0, SD pitch*	0.04	0.01	4.28	**0**.**00**	0.02	0.06	1.04
*HNR*	−0.28	0.06	−5.02	**0**.**00**	−0.39	−0.17	0.75
*Jitter, RAP*	−60.32	37.04	−1.63	0.10	−132.91	12.28	0.00
*Shimmer, APQ3*	−10.04	9.10	−1.10	0.27	−27.87	7.79	0.00
*CPP*	−0.03	0.06	−0.60	0.55	−0.14	0.08	0.97
*LTAS, mean*	−0.01	0.02	−0.20	0.84	−0.05	0.04	1.00
*LTAS, SD*	0.01	0.05	0.22	0.83	−0.09	0.11	1.01
*LTAS, slope*	−0.06	0.04	−1.75	0.08	−0.13	0.01	0.94

**Table 13:** LR acoustic feature contribution towards the classification of trustworthy intent, by speaker ethnicity.

Acoustics	Coef. (β)	S.E.	z	p-value	95% C.I.		Odds Ratio (Exp(β))
					Lower	Upper	
**White ethnic**							
*Duration*	0.26	0.30	0.85	0.40	−0.34	0.85	1.29
*F0, mean pitch*	0.03	0.01	5.11	**0**.**00**	0.02	0.04	1.03
*F0, SD pitch*	0.04	0.01	4.71	**0**.**00**	0.02	0.06	1.04
*HNR*	−0.36	0.06	−6.31	**0**.**00**	−0.48	−0.25	0.70
*Jitter, RAP*	−27.09	35.19	−0.77	0.44	−96.05	41.88	0.00
*Shimmer, APQ3*	−21.41	8.96	−2.39	**0**.**02**	−38.97	−3.86	0.00
*CPP*	−0.11	0.05	−1.99	0.05	−0.21	0.00	0.90
*LTAS, mean*	−0.02	0.02	−1.09	0.28	−0.07	0.02	0.98
*LTAS, SD*	−0.03	0.06	−0.48	0.63	−0.15	0.09	0.97
*LTAS, slope*	−0.06	0.03	−1.76	0.08	−0.12	0.01	0.94
**Black ethic**							
*Duration*	−0.89	0.33	−2.68	**0**.**01**	−1.55	−0.24	0.41
*F0, mean pitch*	0.03	0.01	5.15	**0**.**00**	0.02	0.04	1.03
*F0, SD pitch*	0.02	0.01	2.41	**0**.**02**	0.00	0.04	1.02
*HNR*	−0.34	0.07	−4.65	**0**.**00**	−0.49	−0.20	0.71
*Jitter, RAP*	−98.56	46.27	−2.13	**0**.**03**	−189.25	−7.87	0.00
*Shimmer, APQ3*	−5.06	16.01	−0.32	0.75	−36.43	26.32	0.01
*CPP*	0.00	0.07	0.03	0.98	−0.13	0.13	1.00
*LTAS, mean*	−0.07	0.03	−2.66	**0**.**01**	−0.13	−0.02	0.93
*LTAS, SD*	−0.12	0.06	−2.08	**0**.**04**	−0.24	−0.01	0.89
*LTAS, slope*	−0.03	0.04	−0.89	0.37	−0.11	0.04	0.97
**South Asian ethnic**							
*Duration*	−0.30	0.34	−0.89	0.38	−0.97	0.37	0.74
*F0, mean pitch*	0.02	0.01	3.44	**0**.**00**	0.01	0.03	1.02
*F0, SD pitch*	0.03	0.01	2.70	**0**.**01**	0.01	0.05	1.03
*HNR*	−0.18	0.06	−2.76	**0**.**01**	−0.30	−0.05	0.84
*Jitter, RAP*	−24.57	33.82	−0.73	0.47	−90.85	41.71	0.00
*Shimmer, APQ3*	1.27	11.95	0.11	0.92	−22.15	24.68	3.55
*CPP*	−0.03	0.06	−0.53	0.60	−0.14	0.08	0.97
*LTAS, mean*	−0.02	0.02	−0.87	0.38	−0.07	0.03	0.98
*LTAS, SD*	−0.10	0.06	−1.73	0.08	−0.22	0.01	0.90
*LTAS, slope*	0.05	0.03	1.69	0.09	−0.01	0.11	1.05

### Conclusion

In this paper, a new speech dataset of 1,152 audio recordings from 96 speakers of different ethnicities (white, black, south Asian) and age groups (18–90 years old) was presented; this dataset allows the production of trustworthy intent as perceived by the speakers themselves, in spoken English, to be investigated. The classification of acoustic and spectral features extracted from the audio samples, yielded accuracies of about 70% and AUC values between 71 and 78% for both linear and non-linear classification models (RF and LR). Results suggest that mean F0, SD F0, HNR, CPP and shimmer are the most common and relevant features for discriminating natural speaking voice (i.e. neutral intent) and speech produced with the intent to sound trustworthy across all our speaker demographics. LTAS seems to be the least influential factor, albeit not the case for black ethnicity in LR. Overall, our findings seem to align with and offer additional insights to past research in the field^3,8,30–33^. Further analysis is needed to gain deeper insights into the production, recognition and perception of trustworthiness in spoken language, and this dataset can serve as a good resource to the research community and contribute to future research and insights in this multi-disciplinary area.

## Usage Notes

All data are readily accessible to the public under the terms of a CC-By Attribution 4.0 International license on our OSF repository^17^. We encourage the research community to leverage and appropriately acknowledge this speech audio dataset in their analyses and publications by citing the work mentioned in the README.md file on the OSF repository.

## Data Availability

The Python source code employed to evaluate this dataset is openly accessible on the OSF repository^17^. Please read the README.md file in the repository for more information on how to run the scripts yourself.
